# Taxonomic Identification of Mediterranean Pines and Their Hybrids Based on the High Resolution Melting (HRM) and *trnL* Approaches: From Cytoplasmic Inheritance to Timber Tracing

**DOI:** 10.1371/journal.pone.0060945

**Published:** 2013-04-05

**Authors:** Ioannis Ganopoulos, Filippos Aravanopoulos, Panagiotis Madesis, Konstantinos Pasentsis, Irene Bosmali, Christos Ouzounis, Athanasios Tsaftaris

**Affiliations:** 1 Institute of Applied Biosciences, Centre for Research & Technology Hellas (CERTH), Thessaloniki, Greece; 2 Department of Genetics and Plant Breeding, Aristotle University of Thessaloniki, Greece; 3 Laboratory of Forest Genetics and Tree Breeding, Faculty of Forestry and Natural Environment, Aristotle University of Thessaloniki, Greece; 4 Donnelly Centre for Cellular & Biomolecular Research, University of Toronto, Toronto, Ontario, Canada; Fordham University, United States of America

## Abstract

Fast and accurate detection of plant species and their hybrids using molecular tools will facilitate the assessment and monitoring of local biodiversity in an era of climate and environmental change. Herein, we evaluate the utility of the plastid *trnL* marker for species identification applied to Mediterranean pines (*Pinus* spp.). Our results indicate that *trnL* is a very sensitive marker for delimiting species biodiversity. Furthermore, High Resolution Melting (HRM) analysis was exploited as a molecular fingerprint for fast and accurate discrimination of *Pinus* spp. DNA sequence variants. The *trnL* approach and the HRM analyses were extended to wood samples of two species (*Pinus nigra* and *Pinus sylvestris*) with excellent results, congruent to those obtained using leaf tissue. Both analyses demonstrate that hybrids from the *P. brutia* (maternal parent) × *P. halepensis* (paternal parent) cross, exhibit the *P. halepensis* profile, confirming paternal plastid inheritance in Group Halepensis pines. Our study indicates that a single one-step reaction method and DNA marker are sufficient for the identification of Mediterranean pines, their hybrids and the origin of pine wood. Furthermore, our results underline the potential for certain DNA regions to be used as novel biological information markers combined with existing morphological characters and suggest a relatively reliable and open taxonomic system that can link DNA variation to phenotype-based species or hybrid assignment status and direct taxa identification from recalcitrant tissues such as wood samples.

## Introduction

Forest trees constitute about 82% of continental biomass and harbour more than 50% of terrestrial biodiversity. The first seed plants to have evolved were Gymnosperms, which today occupy about 25% of the planet’s forests. *Pinus* is the most important genus within the Gymnosperms and within the Pinaceae family, taking into account the number of species (109), [1]and their contribution to forest ecosystems [2]. Pine trees (*Pinus* L.) are important elements of the Mediterranean landscape. They have played a major role in the evolution of Mediterranean flora and vegetation [3] and have been widely used by its inhabitants since prehistoric times [4]. The genus is divided into subgenera, sections and subsections. Various classifications have been proposed in this genus [5], [6] with the most recent efforts focusing on DNA phylogenetics to identify related species [7], [8].

Comprehensive classifications of the genus *Pinus* were established earlier using morphological and anatomical traits [6], [9]; later, molecular data were also included [8], [10], [11]. Congruence between classical and molecular taxonomy has been generally observed, nevertheless variations are noticed between classical and molecular approaches. Some of the first molecular studies in pines involved restriction patterns of the plastid genome [12], which is paternally inherited in the genus *Pinus* (e.g. [13]). A number of studies have focused on particular taxa within the genus *Pinus,* for instance in subgenus *Pinus* [14] and Eurasian species [15]. More recent classifications, including a large number of species, were established from nuclear internal transcribed spacer (ITS) [16] and plastid sequences (*rbcL*) [8], [16] while molecular technologies were used to identify natural hybrids, detect introgression and study hybrid speciation [17], [18]. Plastid sequences being uniparentally inherited can be part of a hybrid identification protocol. Unequivocal species and hybrids identification in pines is important for both basic research and applied forestry.

The use of DNA sequences to identify forest tree species and hybrids coupled with advances in wood DNA isolation requiring minute amounts of DNA for PCR-based approaches, offer an innovative opportunity for tracing wood and wood products throughout the chain-of-custody from the forest to the consumer. Wood DNA is stable and cannot be manipulated [19]; therefore the development of reliable and efficient tracing methods can be incorporated in forestry applications. Two early examples of relevant DNA marker applications are the identification of wood origin used for the production of wine barrels in France [20] and the DNA fingerprinting of rootstock and logs of the endangered tropical species *Intsia* spp. (Merbau) to control and restrain illegal logging [21]. To our knowledge there has not been a pertinent systematic study using these approaches with regard to *Pinus* spp. yet.

The *trnL* approach is a method of identifying plant species using short DNA sequences [22], [23]. The method is extremely useful in species identification, including cryptic species, biodiversity studies, forensic analysis and phylogenetics. Different short length regions of the plastid genome have been used as DNA molecular identification sites primarily for species identification [22], [23]. The CBOL (Consortium for the Barcode of Life) plant working group recommended using the 2-locus combination of *rbcL*+*matK* as a plant barcode [22]. Nevertheless, [24] had already shown the suitability of the *trnL* intron as a barcode for plants due to its well analyzed and thoroughly understood evolutionary patterns, conserved secondary structure, significant discrimination power, availability of universal primers and wide application. The use of *trnL* (UAA) intron as a supplementary locus has been advocated for those projects that involved PCR amplification of DNA from highly degraded tissues such as wood [25]. In this communication, we consider the *trnL* locus as a DNA identification marker. Molecular identification using plastid DNA regions have been extended already in the food industry, evolution studies and forensics [26], [27], [28], [29]. In gymnosperms, the *trnL* approach has been applied in sparse samples of Cycadales (cycads) and Pinales (conifers) [5], [30], [31]. Gymnosperm life history characteristics (incomplete reproductive isolation, primarily paternal plastid inheritance, large effective population sizes, long generation cycles; [32]) complicate the use of the *trnL* approach despite a relatively restricted number of extant species (<1000).

The typical *trnL* approach is time consuming and dependent upon highly experienced personnel and considerable resources, which can hinder large-scale application for forest tree populations. High-resolution melting (HRM) analysis [33] allows genotyping and fingerprinting by discriminating DNA sequence variants such as single nucleotide polymorphisms (SNPs) and small insertion and deletions (indels) based on the shape of melting transitions (Tm) of real-time PCR products [33], [34], [35]. HRM analysis can be applied not only for allele discrimination by targeting well-characterized SNPs, but also for screening of the existence of unknown sequence variations without a sequencing process. HRM is a powerful and accurate technique which is cheaper, faster and simpler than alternative approaches requiring post-PCR processing enzyme restriction and electrophoresis, labelled probes for SNP detection sequencing or TaqMan-probe-based real-time PCR [36]. HRM has been used as a molecular diagnostic for species discrimination in higher plants [27], [37], [38] including trees and shrubs, such as *Prunus* [37] and *Vaccinium* [39].

The objectives of the present study were as follows: (a) to analyze sequence variation of the *trnL* plastid region and test its usefulness in the identification of Mediterranean *Pinus* species (including the endemic to Transcaucasia but introduced to the eastern Mediterranean *P. eldarica*), (b) to develop a rapid, simple, and stable HRM real-time PCR assay targeting the *trnL* region for *Pinus* species identification as an alternative and efficient approach that can be used in the molecular taxonomy studies of pines, (c) to apply HRM analysis of the *trnL* plastid region for the investigation of plastid inheritance using interspecific hybrids of Group Halepensis (*P. halepensis* and *P. brutia*) as an case study, and (d) to trace wood origins by HRM analysis for the highly commercial pine species *P. sylvestris* and *P. nigra*.

## Materials and Methods

### Ethics Approval

No specific permissions were required for sampling from the Aristotle University Botanic Garden for which there is open access for members of the Faculty of Forestry and Natural Environment of the University (in this case Professor Aravanopoulos F., co-author). Furthermore, no specific permissions were required for sampling from the Aristotle University Laboratory of Forest Genetics and Tree Breeding, Experimental Plantation in Triadi, Greece for which there is open access for members of the Laboratory (in this case Professor Aravanopoulos F., co-author). No specific permits were required for the described field studies. The locations are not privately-owned or protected in any way, and the field studies did not involve endangered or protected species.

### Plant Material and DNA Isolation

Plant material (needle and wood samples) was obtained from the Aristotle University of Thessaloniki Botanic Garden [40], from the Aristotle University of Thessaloniki Forestry Herbarium (TAUF) (http://sciweb.nybg.org/science2/IndexHerbariorum.asp) and the Triadi experimental plantation, of the Laboratory of Forest Genetics and Tree Breeding, Aristotle University [41]. These are located in the greater Thessaloniki area, Greece (Table 1). The *P. brutia x halepensis* interspecific hybrids are full-sib F_1_ hybrids: interspecific hybridization success has been verified by isoenzyme species-specific genetic markers [41]. We have analysed eight samples per pure species originating from different natural populations and 10 samples of the full-sib F_1_ hybrids.

**Table 1:** Taxonomical inference, sample origin, code of voucher specimen, collector, place of Voucher deposition and associated references of the Pinus species and hybrids employed in this study.

Taxa	Subgenus	Section	Subsection	Reference	Sample Origin	Voucher Specimen	Collector	Place of Voucher Deposition	Reference
*Pinus brutia* Ten.	*Pinus*	*Pinus*	*Halepenses* Van der Burgh	[9]	Aristotle University Botanic Garden and TAUF	Pb01-05.100311; Pb06-08.011012	FA Aravanopoulos	FGL-AUTh	[41]
*Pinus brutia* x *halepensis*	*Pinus*	*Pinus*	*Halepenses* Van der Burgh	[73]	Triadi Experimental Plantation	Pbh01-10.120311; Pbh06-08.011012	FA Aravanopoulos	FGL-AUTh	[40]
*Pinus eldarica* Medwed.	*Pinus*	*Pinus*	*Halepenses* Van der Burgh	[6]	Triadi Experimental Plantation	Pe01-05.120311; Pe06-08.011012	FA Aravanopoulos	FGL-AUTh	[40]
*Pinus halepensis* Mill.	*Pinus*	*Pinus*	*Halepenses* Van der Burgh	[9]	Aristotle University Botanic Garden and TAUF	Pha01-05.100311; Pha 06-08.011012	FA Aravanopoulos	FGL-AUTh	[41]
*Pinus heldreichii* Christ	*Pinus*	*Pinus*	*Pinus*	[9]	Aristotle University Botanic Garden and TAUF	Phe01-05.100311; Phe06-08.011012	FA Aravanopoulos	FGL-AUTh	[41]
*Pinus nigra* Arnold	*Pinus*	*Pinus*	*Pinus*	[9]	Aristotle University Botanic Garden and TAUF	Pn01-05.100311; Pn06-08.011012	FA Aravanopoulos	FGL-AUTh	[41]
*Pinus peuce* Griseb.	*Strobus* Lemm	*Strobus*	*Strobi* Loud.	[9]	Aristotle University Botanic Garden and TAUF	Ppe01-05.100311; Ppe06-08.011012	FA Aravanopoulos	FGL-AUTh	[41]
*Pinus pinea* L.	*Pinus*	*Pinus*	*Pineae* Little and Critchfield	[9]	Aristotle University Botanic Garden and TAUF	Ppi01-05.100311; Ppi06-08.011012	FA Aravanopoulos	FGL-AUTh	[41]
*Pinus sylvestris*	*Pinus*	*Pinus*	*Pinus*	[9]	Aristotle University Botanic Garden and TAUF	Psy01-05.100311; Psy06-08.011012	FA Aravanopoulos	FGL-AUTh	[41]

FGL-AUTh: Laboratory of Forest Genetics and Tree Breeding, Aristotle University of Thessaloniki, TAUF: Aristotle University of Thessaloniki Forestry Herbarium.

DNA isolation from fresh tissue was performed using 0.1 g of pine needle as starting material in the form of fine powder by employing the Qiagen DNeasy plant mini kit according to manufacturer’s instructions. DNA concentration was estimated by standard spectrophotometric methods at 260 nm and 280 nm UV lengths using an Eppendorf BioPhotometer. DNA integrity was tested by gel electrophoresis in a 0.8% agarose gel. Samples were then diluted to a 20 ng/µL concentration.

DNA from wood was extracted using the DNeasy Plant Mini Kit (Qiagen) and applying the same modifications and optimizations as reported elsewhere [40]. Prior to extraction, the surface tissues of wood samples were removed using a saw to avoid contamination with other plant DNA. For DNA extraction, 50–100 mg of shavings produced by drilling of the clean inner part (sapwood) of wood samples was used. We used 80–90 mg of wood shavings for each experiment. Polyvinylpyrolidone (PVP40000, Roth) was added into 800 µL of the AP1 lysis buffer from Qiagen (Hilden, Germany; step up to 2.6% w/v). For all samples, 800 µL AP1 lysis buffer was added to the ground wood samples instead of 500 µL because of sample high absorption capacity. The mixture was incubated overnight at 65°C under a 60 rpm vertical rotation [42], [43]. Blank control extractions were performed simultaneously, starting out with an empty reaction tube and were treated identically for the rest of the analysis. We used individually isolated triplicate samples for all species.

### Verification of the DNA Isolation Method

DNA samples isolated from pine needles and wood from the same tree were analyzed by PCR amplification, HRM genotyping and sequencing. PCR reactions were prepared under a separate PCR using a dedicated set of pipettes. Extraction and PCR set-up were performed on different days. Blank PCR reactions were performed by adding the appropriate amount of sterile ultra-pure water to the reaction [43].

### PCR Amplification for *trnL* Analysis

PCR amplification was performed in a total volume of 25 µL in a MJ research thermocycler. The reaction mixture contained 20 ng genomic DNA, 1X PCR buffer, 2.5 mM MgCl_2_, 0.2 mM dNTP, 300 nM forward and reverse primers (Table 2), and 0.5 U Kapa *Taq* DNA polymerase (Kapa Biosystems, USA). Initial denaturing step of 95°C for 3 min followed by 30 cycles of 95°C for 20 s, 54°C for 40 s and 72°C for 40 s, then a final extension step of 72°C for 2 min.

**Table 2:** Primers used in the *trnL* approach and the HRM analysis of eight *Pinus* spp. used in this study.

Region	Primer	Tm	Feature	Reference
trnL1- F	5-CGAAATCGGTAGACGCTACG-3	59°C	Sequence	[69]
trnL1-R	5-GGGGATAGAGGGACTTGAAC-3	59°C	Sequence	[69]
trnL2-F	5-GGGCAATCCTGAGCCAA-3	60°C	HRM	[69]
trnL2-R	5-CCATTGAGTCTCTGCACCTATC-3	55°C	HRM	[69]
PinustrnLHyb-F	5-GACTCTATCTTTATCCTCGTCC-3	60°C	HRM	This study
PinustrnLHyb-R	5-GGTCCAATACTGTAGTTATAGAAC-3	60°C	HRM	This study
PinustrnLWood-F	5-CTTATGAATAAAATGCTTGGAACG-3	60°C	HRM	This study
PinustrnLWood-R	5-ATAACATCAGACAAAACTGG-3	60°C	HRM	This study

### PCR Amplification for HRM Analysis

The primers used are presented in Table 2. PCR amplification, DNA melting and end point fluorescence level acquiring PCR amplifications were performed in a total volume of 15 µL on a Rotor-Gene 6000 real-time 5P HRM PCR Thermocycler (Corbett Research, Sydney, Australia). A third generation DNA intercalating dye, Syto®-9, which at high concentrations can saturate all available sites within double stranded DNA, was used. The reaction mixture contained 20 ng genomic DNA, 1X PCR buffer, 2.5 mM MgCl_2_, 0.2 mM dNTP, 300 nM forward and reverse primers (Table 2), 1.5 mM Syto®-9 green fluorescent nucleic acid stain and 0.5 U Kapa *Taq* DNA polymerase (Kapa Biosystems, USA). Syto®-9 fluorescence provides a more accurate assessment of DNA melting status compared to SYBR Green I and can be used to monitor the accumulation of the amplified product during PCR and the subsequent product melting on the RotorGene 6000 (software version 2.0.2, Corbett Life Science, Cambridge, UK).

A rapid PCR protocol was conducted in a 36-well carousel using an initial denaturing step of 94°C for 3 min followed by 30 cycles of 95°C for 20 s, 54°C for 30 s and 72°C for 40 s, then a final extension step of 72°C for 2 min. The fluorescent data was acquired at the end of each extension step during PCR cycles. Before HRM, the products were denatured at 95°C for 5 s, and then annealed at 50°C for 30 s to randomly form DNA duplexes. Specifically, to trace the origin of wood with HRM we used the DNA sequence information obtained for *trnL* (Figure S1) and we designed specific primers (Table 2) which can discriminate DNA samples between *P. sylvestris* and *P. nigra* by a 1 bp polymorphism. The PCR products were separated by electrophoresis in 3.5% agarose gel (not shown).

HRM was performed as follows: pre-melt at the first appropriate temperature for 90 s, and melt at a ramp of 10°C in an appropriate temperature range at 0.1°C increments every 2 s. The fluorescent data were acquired at the end of each increment step. End point fluorescence level was acquired following the melting process by holding at 60°C for 5 min. In order to further increase the reproducibility and reliability of the HRM curve analysis (by obtaining similar amplified quantities of final PCR products before melting), finer adjustments by diluting were made to the genomic DNA templates obtained from needles and woods of each of the species. A Ct parameter of 22±4 cycles at a threshold of 0.01 of the normalized fluorescence was established. All samples were examined in duplicate.

### Identification of PCR Products HRM Analysis

The Rotor-Gene 6000 proprietary software (vs. 2.0.2) was used to genotype species, subspecies and hybrids. The negative derivative of fluorescence (F) over temperature (T) (dF/dt) curve primarily displaying the Tm, the normalized raw curve depicting the decreasing fluorescence vs. increasing temperature, and difference curves [33] were primarily used. Furthermore, a two-step procedure was followed [38] to assess similarity of unknown HRM curves with a known one. Each species was set as a ‘genotype’ (reference species) and the average HRM Genotype Confidence Percentages (GCPs) (value attributed to each species being compared to the genotype, with a value of 100 indicating an exact match) for the replicates (disregarding the most outlying replicate) were tabulated [44]. GCPs were re-coded from a 1–100 to a 1–20 range of values to decrease the number of different genotypes causing small differences in the shape of the melting curves giving slightly different GCPs [37]. The means of the confidence percentage of the species replicates assigned to a representative genotype, together with the standard deviation, were thus obtained. In order to assess whether *Pinus* species would theoretically be distinguishable from each other corresponding sequences of *trnL* amplicons from all eight *Pinus* species were analysed for their melting pattern using the computer program uMELT [45].

### Sequence Analysis

PCR products were directly sequenced in two directions for each product with Big Dye terminator v3.1 Cycle sequencing kit (PE Applied Biosystems, Foster City, CA, USA) in an automated ABI 3730 sequencer (PE Applied Biosystems). The sequences were aligned with the MAFFT multiple sequence alignment web service implemented in JalView 2.6.1 [46].

### Data Analysis

The sequence character-based method [47] was used with DnaSP [48] and the information from each site was treated as a character to distinguish the species. Assembly of the final sequences was performed using the combination of Phred/Phrap/Consed software (http://www.phrap.org/). Sequence quality was determined by Phred base caller [49]. Bases with Phred quality scores less than 30 were manually inspected and edited where appropriate using Consed [50]. The B-index cut-off has been set according to [51].

## Results

### Barcoding of PCR Products by HRM Analysis

The amplicons of oligonucleotide *trnL* sets were subjected to HRM curve analysis. Optimum ramps for this oligonucleotide set were selected based on the highest confidence percentages of the normalized curves and the ability to visually distinguish between the conventional melt curve peaks obtained from each reference species. The optimum ramp was 0.1°C s^−1^ for the *trnL* oligonucleotide set.

**Figure 1:**
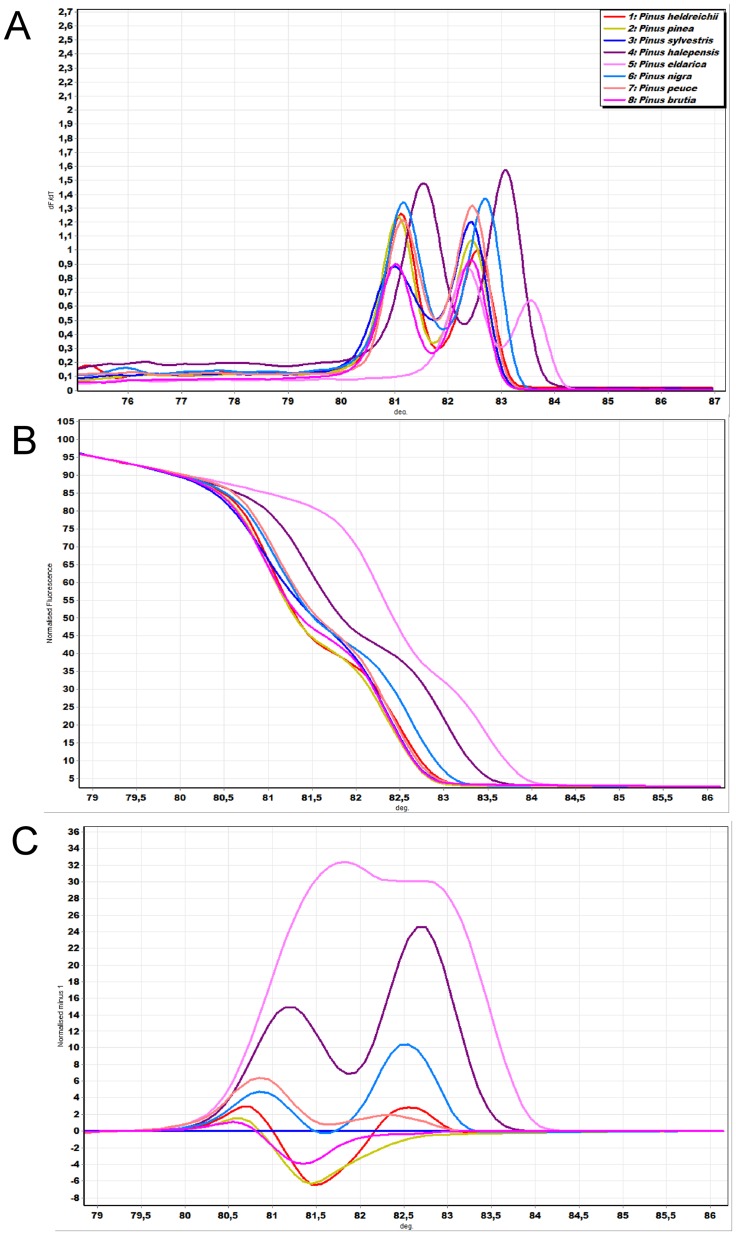
Molecular identification of *Pinus* species using HRM analysis with the *trnL* plastid marker. (A) Melting peaks of *trnL* amplicons of eight *Pinus* species (B) Conventional melting curves of amplicons from eight *Pinus* species generated using the universal *trnL* marker at a ramp of 0.1°C s^−1^. (B) Difference graph of eight species using *Pinus sylvestris* as genotype. Assigned genotypes using a cut off confidence value of 90%. The HRM of all other species were compared to this control and resulted as *Pinus sylvestris* at ≥90% confidence or as variation if <90% confidence.

To investigate whether the polymorphism in the *trnL* region of different *Pinus* species was detectable in conventional melting curves, DNA melting profile analysis was performed. Figure 1A–C depicts the melting peaks (and DNA melting curves) generated for each *Pinus* species. The peaks represent the temperatures at which the maximal fluorescence decay occurs and are indicative of the dissociation temperature of the amplified product(s). The melting peaks temperature for all pine species are presented in Table 3. Peaks were evident for each species within the range 80.98°C to 83.55°C. All species profiles produced two maxima.

**Table 3:** Mean ± standard deviation (SD) of the points for the melting peaks of the amplicons resulted from the eight *Pinus* species in several runs of *trnL* PCR followed by high resolution melt curve analysis at a ramp of 0.1^o^ s^−1^.

Species	Peak 1	Peak 2
	(°C) ± SD	(°C) ± SD
*P. brutia*	81.02±0.1	82.43±0.3
*P. brutia x halepensis*	81.53±0.1	83.07±0.1
*P. eldarica*	82.35±0.2	83.55±0.2
*P. halepensis*	81.53±0.1	83.07±0.1
*P. heldreichii*	81.12±0.1	82.55±0.2
*P. nigra*	81.15±0.2	82.7±0.2
*P. peuce*	81.15±0.3	82.45±0.1
*P. pinea*	81.05±0.3	82.45±0.3
*P. sylvestris*	80.98±0.2	82.43±0.2

Analysis of the normalized HRM curves with the marker *trnL* (Figure 1A, B) revealed that most of the species could be distinguished visually, for example, *P. halepensis* and *P. nigra,* as the HRM curves obtained are highly characteristic for each amplicon. Despite the fact that some species peaks present overlapping, the HRM melting curves are quite characteristic for each species based on shape, being dependant on the interplay between GC content, length of amplified product and sequence, even when they define the same *T*_m_ values. Furthermore, closer examination of the HRM difference curves, with the mean *P. pinea* curve as the baseline, revealed part of the curve sitting outside the 90% confidence interval curve, suggesting that all the examined taxa via the HRM curves are different species (Figure 1A) based on the samples analyzed. Assigning species *P. sylvestris* as a reference genotype we were able to estimate the confidence value of similarity between *P. sylvestris* and the other *Pinus* species used in the study. This was achieved by subtracting the area of *P. sylvestris* melting curve (difference graph, Figure 1B) from the rest of the produced melting curves by the other species. We have showed that *trnL* was a potential region for distinguishing the species studied (Figure 1B). The average genotype confidence percentages (GCPs) resulting from HRM analysis of the *trnL* region of eight pine species are shown in Table 4. Moreover, GCPs were calculated and a cut off value of 90% was used to assign a genotype for the *trnL* region. The highest GCP (88.83) was found between the *P. pinea* and *P. brutia* species, while the lowest (0.0) was between *P. eldarica* and all other *Pinus* species.

**Table 4:** Average genotype confidence percentages (±3.21) resulting from HRM analysis of the universal plastid region *trnL* of eight *Pinus* species examined at a ramp of 0.1°C.

Species	*P. brutia*	*P. brutia x halepensis*	*P. eldarica*	*P. halepensis*	*P. heldreichii*	*P. nigra*	*P. peuce*	*P. pinea*	*P. sylvestris*
*P. brutia*	100								
*P. brutia x halepensis*	0.04	100							
*P. eldarica*	0	0.07	100						
*P. halepensis*	0.04	99.53	0.07	100					
*P. heldreichii*	78.52	0.07	0	0.07	100				
*P. nigra*	28.7	3.04	0	3.04	42.85	100			
*P. peuce*	57.74	0.42	0	0.42	54.35	52.44	100		
*P. pinea*	88.83	0.02	0	0.02	84.85	21.79	45.25	100	
*P. sylvestris*	87.38	0.11	0	0.11	61.52	35.05	70.01	69.61	100

### Plastid DNA Inheritance with the DNA-barcoding and HRM Approaches

**Figure 2:**
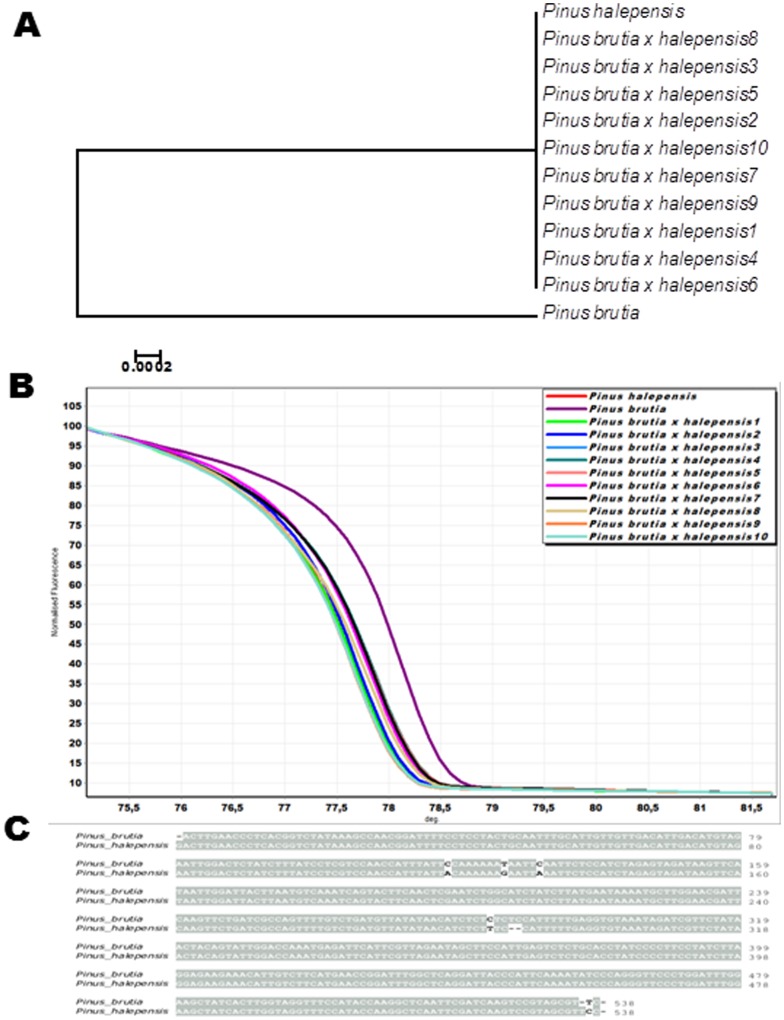
*trnL* approaches for cytoplasmic inheritance. (A) The dendrogram relations of *Pinus brutia, P. halepensis*, and their F_1_ hybrids based on the cpDNA *trnL* sequence. (B) Normalized high resolution melt curves of *trnL* amplicons generated from *Pinus brutia, P. halepensis*, and their F_1_ hybrids using the oligonucleotide set Ph-trnL. (C) Comparison of the partial nucleotide sequence of the *trnL* from *Pinus brutia* and *P.halepensis*.

Partial sequences of the gene plastid *trnL* revealed differences in the nucleotide composition between *P. halepensis* and *P. brutia*. To confirm the direction of parental inheritance, paternal relationships between the two parental species and their hybridized offspring were constructed. The NJ tree of the plastid *trnL* indicated that the F_1_ hybrids were grouped together with *P. halepensis* (Figure 2A). The exclusive presence of the paternal parent’s *P. halepensis* sequence in progeny hybrids indicates the paternal inheritance of cpDNAs in these Group Halepensis species.

HRM analysis of the *trnL* region detected two different genotypes corresponding to *P. halepensis* and *P. brutia* (Figure 2B). All artificial *P. brutia* x *halepensis* hybrids had the same genotype as their paternal parent *P. halepensis*. These results also confirmed the paternal inheritance of plastid DNA in the Group Halepensis species. Furthermore, the HRM results were verified through sequencing of the PCR amplification products for the *trnL* intron region (Figure S1). Although the sequences are well conserved, we have identified mainly single nucleotide polymorphisms. In addition, we have detected two insertions (CCAGT in *P. peuce* and TC in *P. eldarica* and *P. brutia*), as well as one ATTCA deletion in *P. nigra* and *P. sylvestris*.

### *Pinus* Identification with the *trnL* Marker

We obtained 74 *trnL* sequences in total from eight different *Pinus* species (Table S1) and 10 artificial *P. brutia* x *halepensis* hybrids. All sequences have been deposited in NCBI database (Table S1). A very good amplification success was evident in all species studied. The analyzed loci exhibited high PCR success with *trnL* primers reaching a success rate of 99.8%. All PCR products corresponding to the *trnL* DNA marker were successfully sequenced and high quality bidirectional sequences were obtained. Sequence quality was very high as determined by the Phred base caller and Consed. The B-index cut-off has been set to 0.89.

The *trnL* matrix presented a sequence of 549 bp and indels with an average length of 3.714 bp. The average number of indels was 9.09. The mean sequence divergence in *Pinus* species was 1.390. The discriminating power of the *trnL* marker at the species level was 100%.

To establish character-based molecular identification sites for all *Pinus* species studied, eight nucleotide positions of the *trnL* nuclear region were chosen. These particular nucleotide positions revealed the highest numbers of diagnostic characters (Table 5). Using only these chosen positions, all species could be distinguished by at least one diagnostic site. *P. brutia* and *P. eldarica* in particular, shared similar first peaks in both uMELT and HRM curve analyses, but nevertheless the position of the second peaks differed, making them distinct from one another. Overall, we identified eight diagnostic positions at sites 69, 115, 122, 130, 35, 140, 298 and 547 (Table 5). They included all Character Attributes (CAs) for closely related sequences such as *P. brutia* and *P*. *eldarica*, and further sites with a high number of CAs (i.e., more than one clade had a unique character state or polymorphism at that site). *P. brutia* differed only at one diagnostic site from *P. eldarica*.

**Table 5:** Character-based DNA identities for eight *Pinus* species and one interspecific hybrid for the *trnL* plastid region.

Position								
Species	69	115	122	130	135	140	298	547
*Pinus brutia*	g	a	c	t	c	c	g	c
*Pinus brutia x halepensis*	g	a	a	g	a	c	g	c
*Pinus eldarica*	g	a	c	t	c	c	g	t
*Pinus halepensis*	g	a	a	g	a	c	g	c
*Pinus heldreichii*	g	t	c	g	c	c	t	–
*Pinus nigra*	t	a	c	t	c	c	g	t
*Pinus peuce*	g	a	c	t	c	t	g	c
*Pinus pinea*	g	t	c	g	c	t	g	–
*Pinus sylvestris*	t	a	c	t	c	c	g	c

Diagnostic character states at eight selected nucleotide positions for *trnL*, different in at least one position per species combination, are shown.

### Timber Tracing with HRM Analysis

**Figure 3:**
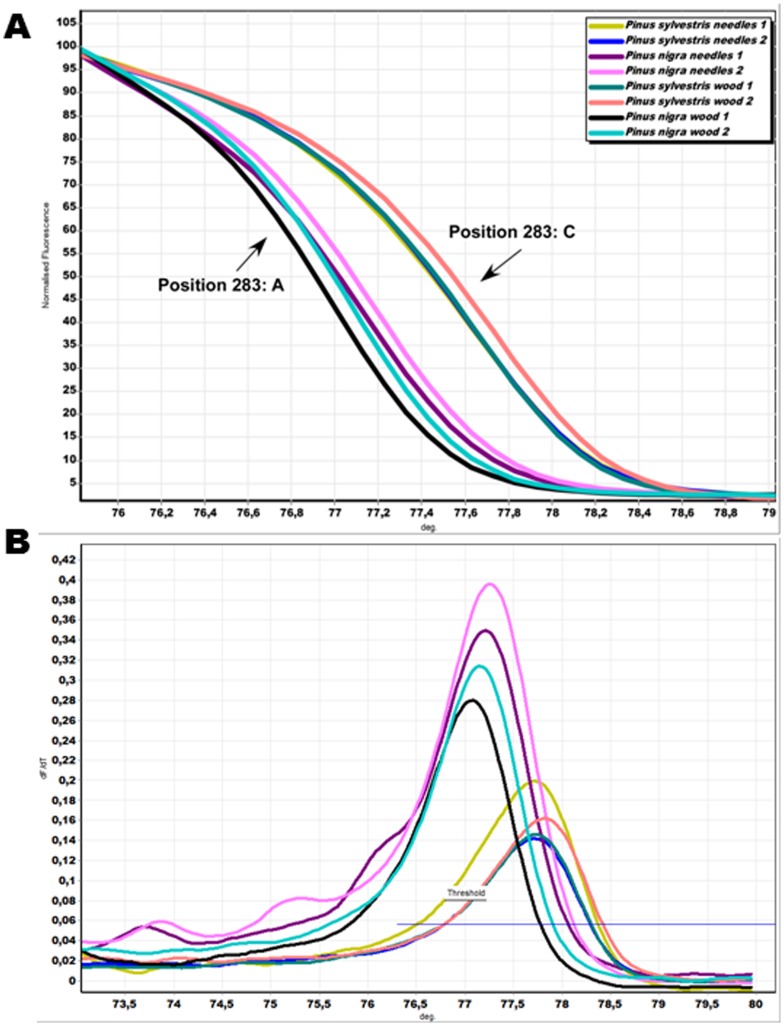
HRM analysis for timber tracing. (A) Timber tracing with HRM of two *Pinus nigra* and *P. sylvestris* with the specific *trnL* plastid marker. (B) Conventional melt curves of the specific *trnL* marker. Color codes referring to the genotypes used are presented in the upper part of Figure 3A.

In order to test the DNA isolation method, needle and wood DNA extracts from the same tree were amplified and genotyped with a *Pinus* specific plastid *trnL* primer. In addition, no amplification product was noted both for isolation and for PCR negative controls throughout the whole study. After the confirmation that single species can be identified by HRM analysis, we applied the same approach for species identification using fresh wood samples. We report herein results from two species (*P. nigra* and *P. sylvestris*), although similar results were obtained from all *Pinus* species studied, as well as from old wood specimens (Ganopoulos *et al*., in preparation). Figures 3A and 3B depict the HRM melting peaks and curves of wood and needles that originated from the same tree have the same profile. The normalized HRM curves for the amplicons of the two wood products (one per individual tree and corresponding species), based on HRM analysis with the *trnL* marker PinustrnL2 are shown (Figure 3A). Each genotype produced a unique melting plot that was easily distinguishable from the other and consistent with the observed nucleotide differences among them. The melting profile of the *trnL* PCR amplicon from *P. sylvestris* produced a single melting peak, whereas melting of the *P. nigra* amplicon produced an additional very distinct peak (Figure 3A). The melting profiles within species were consistent in all samples and replicates. The results show that the *trnL* sequence obtained from wood and needles of the same tree were identical and the sequences differed among *P. sylvestris* and *P. nigra* in one position (283: C>A) allowing species discrimination.

## Discussion

### *Pinus* Identification with the *trnL* Marker and HRM Analysis

The PCR amplification success rate was effective since there was no apparent relation between amplicon size and PCR success rate. An increase in amplicon size may result in an adverse effect on the PCR amplification success rate [19]. However, such effects were not observed, potentially due to the relatively short fragment length of the *trnL* region (80 bp) used. We have demonstrated that sequence variation within the analyzed region of the *trnL* gene enabled the use of HRM analysis for the differentiation of even closely related *Pinus* species. The eight samples used originated from different natural populations, presented the same haplotype and showed with statistical confidence the absence of intraspecific variation. Nevertheless, the above results should be further verified by future analysis of a larger sample size both in terms of species, populations and individuals within populations. Unknown samples could be analyzed using the above standard species samples as reference controls. Unknown samples may be analyzed in the absence of reference controls by the comparison of the unknown and reference samples HRM curves (e.g. evaluation of the difference curve GCP values, ΔMelt, or its derivatives). This is possible as HRM curves can be exported in the form of numerical data (Table S2) and imported in analysis programs (such as Microsoft Excel), thus permitting the comparison of the results from different laboratories. Nevertheless, this procedure should be adopted with caution as results may be influenced by several factors such as DNA quantity and quality, *Taq* polymerase, PCR buffer, chromophore used, template quantity and quality and finally the instrument used.

The range of *trnL* amplicons in size (536–542 bp) and variation (1–5%) in nucleotide sequence (not shown) are in agreement with previous studies, in which featured amplicons of 400–500 bp in size that differ by only 1 bp could be consistently differentiated by HRM curve analysis [52], [53]. The HRM method is a closed tube post-PCR method, which permits the rapid analysis of genetic variation in *Pinus* species via the use of plastid molecular identification regions. HRM measures the rate of double stranded DNA dissociation to single stranded DNA with increasing temperature [54]. This gradual denaturation of PCR amplicons is monitoring real-time subtle changes in fluorescent signal over temperature by including a fluorescent dye in the PCR reaction that intercalates homogenously into DNA and fluoresces when bound to dsDNA [55]. The change in fluorescence measures the thermally-induced DNA dissociation by HRM and the observed melting behaviour is characteristic of the particular DNA product as determined by sequence length, GC content, complementarity, and nearest neighbour thermodynamics [54]. The latest progress of this technique enabled the increased resolution and precision of the instruments and the development of saturating DNA dyes, thus allowing the use of HRM for genotyping. Differentiation down to genus and - in many cases - species level is possible based on melting temperatures (Tm) of specific PCR products [56]. HRM analysis has already been used for the identification of other plant species [37], [57], [58]. In our case, accurate melting curves were generated, allowing us to determine whether different amplicons have the same or different sequence [33]. This study has capitalized on the HRM advantage that melting curves from different amplicons can be differentiated on the basis of shape, even when they define the same T_m_ values [59].

The discrimination of Mediterranean *Pinus* spp. based on the sequenced portion of the plastid *trnL* gene and HRM analysis was effective in depicting the taxonomic status of the species employed. Taxa concordance to species was apparent. The sequence variation between taxa also resulted in the differing shapes of the HRM melting curves that separated clearly at all taxonomic levels. Results provide supporting evidence towards the notion of *P. brutia* being a separate species, not a subspecies of *P. halepensis* (*P. halepensis* subsp. *brutia* Ten.) as the current botanic designation holds [60]. This outcome is in agreement to earlier results providing evidence regarding the separate species status of *P. brutia* based on nuclear (isoenzyme; [41], [61]) and cytoplasmic (cpDNA; [62], [63]) genetic markers. Hence, the pertinent section of Flora Hellenica [60]may need to be re-examined. The species status of *P. eldarica* is less clear. This taxa has been considered as a subspecies of *P. brutia* (*P. brutia* subsp. *eldarica* Medwed; [64]), or a separate species [6]. Even though differentiation at the species level by a single diagnostic site has been reported in the literature [65] the differentiation of *P. eldarica* in our study is less apparent than that observed among other *Halepensis* species studied.

### Plastid DNA Inheritance

Taking advantage of the *trnL* polymorphisms and the capacity of the HRM analysis, we were able to confirm the paternal inheritance of the plastid genome in one artificial interspecific *P. brutia x halepensis* cross. Results are in concordance to the initial preliminary results by [62] who used the plastid *matK* gene and a small portion of the 3′-flanking region within the *trnK* intron. The combination of universal primers and high polymorphism of the *trnL* region reported herein is very useful for the study of cytoplasmic inheritance and the identification of closely related species. For instance, the parental inheritance patterns of the plastid genomes of shortleaf pine (*P. echinata* Mill.), loblolly pine (*P. taeda* L.) and slash pine (*P. elliottii* Engelm.) through the *trnL–trnF* intergenic spacer polymorphism analysis was investigated and confirmed [66]. The parental inheritance of cpDNA in conifers has been verified in many species and is widely accepted today as a rule [67].

### Timber Tracing with HRM Analysis

DNA extraction and isolation from fresh wood samples and subsequent species identification by the *trnL* approach and HRM analysis were entirely successful. The corresponding analysis of leaf DNA from each individual showed identical results, therefore indicating that wood can be a reliable DNA source. Especially the success of HRM analysis provides an efficient and reliable tool that greatly facilitates wood identification by simple molecular means. In this respect, HRM analysis further advances wood DNA analysis and extend previous works [68], [69]. Therefore, HRM analysis can be very useful in tracing wood origins, for example regarding illegal logging where logs can be traced back to stumps at least at the species level.

### Conclusions

This is the first comprehensive study describing the application of HRM curve analysis for differentiation of *Pinus* species and hybrids in leaf and wood samples. One *trnL* marker was able to differentiate among the eight *Pinus* species, following HRM curve analysis (Figure1A, 1B). The *trnL* region may not be regarded as the most variable region in the plastid genome [70]. Nevertheless, it has been proved a suitable region for molecular identification based on the plastid genome [24] and this study points towards its application as a candidate marker. The usefulness of this approach is demonstrated by its successful application in the identification [71], [72] of even closely related species including pines [8]. Besides basic research in phylogeography, taxonomy and evolution, the unambiguous and straightforward *Pinus* species identification has multi-faceted practical applications. The presence of species-specific profiles permits the unequivocal identification of natural interspecific hybrids and monitoring in hybrid seed orchards, for instance in *P. brutia x halepensis*. It also permits the evaluation of the success of artificial pollinations, for example in *P. nigra x sylvestris* and *P. brutia x halepensis* hybrids. Forest seed certification, dispersal of forest reproductive material and management of seed source storage and deployment can thus be greatly facilitated. Forensic applications (i.e. in archaeology) can also be advanced through the application of this approach. These results have been extended to the use of wood as a DNA source coupled with the advantages of the application of HRM analysis. Timber tracing with HRM analysis is expected to contribute to future forest certification schemes, forest industry product management, state agencies monitoring, customs offices, illegal trading and false marketing information.

## Supporting Information

Figure S1 **DNA sequence alignment analysis of pine species shows differences in the DNA level.**
(TIF)

Table S1 **GenBank accession code of samples subjected to the species identification test using the** ***trnL*** **region.**
(DOC)

Table S2 **Export raw data of HRM analysis of** ***trnL*** **region.**
(XLS)
