# Supplementary figures and images for: Taxonomic Identification of Mediterranean Pines and Their Hybrids Based on the High Resolution Melting (HRM) and trnL Approaches: From Cytoplasmic Inheritance to Timber Tracing
Source: PLoS One. 2013 Apr 5;8(4):e60945. doi: 10.1371/journal.pone.0060945 (PMC3618329; doi:10.1371/journal.pone.0060945)

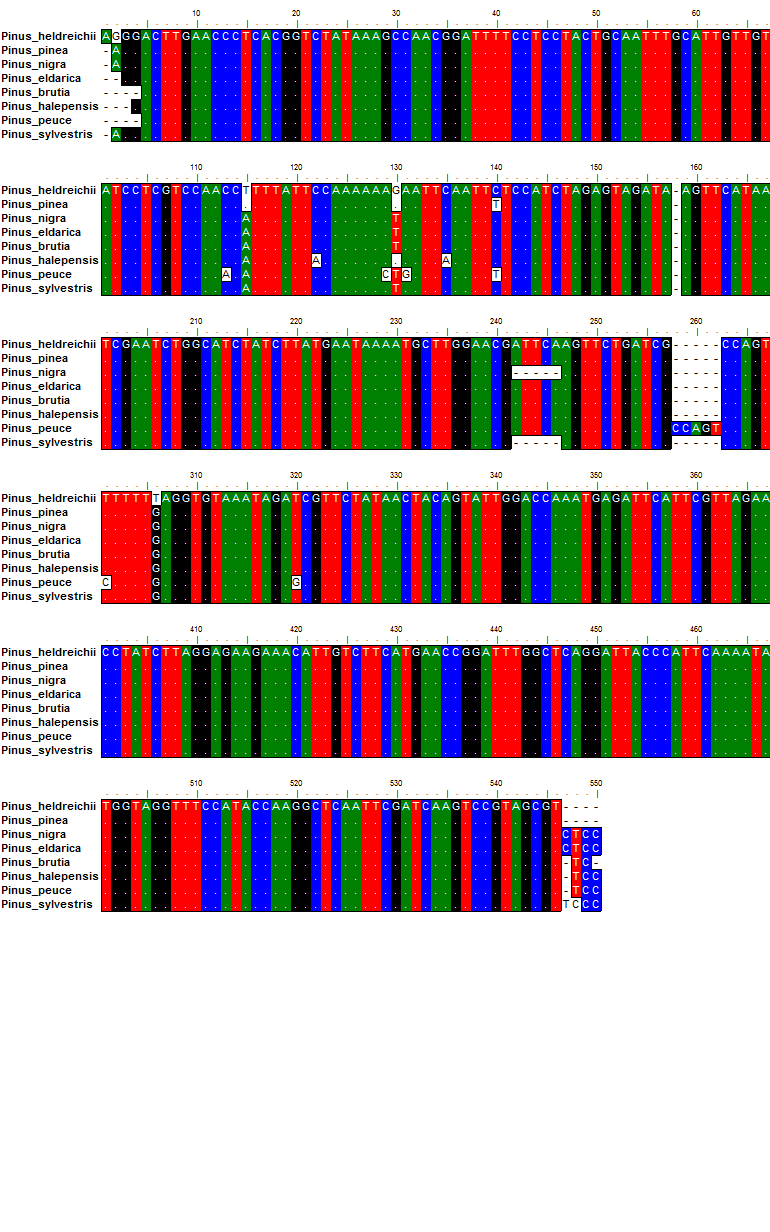

Supplement: Figure S1 — DNA sequence alignment analysis of pine species shows differences in the DNA level. (TIF) [file pone.0060945.s001.tif]
