# Supplementary material for: Taxonomic Identification of Mediterranean Pines and Their Hybrids Based on the High Resolution Melting (HRM) and trnL Approaches: From Cytoplasmic Inheritance to Timber Tracing
Source: PLoS One. 2013 Apr 5;8(4):e60945. doi: 10.1371/journal.pone.0060945 (PMC3618329; doi:10.1371/journal.pone.0060945)
Supplement: Table S1 — GenBank accession code of samples subjected to the species identification test using the trnL region. (DOC) [file pone.0060945.s002.doc]

Table S1: GenBank accession code of samples subjected to the species identification test using the *trnL* region

| Species | Voucher code | Genbank accession code *trnL* |
| --- | --- | --- |
| *Pinus heldreichii* | Phe01.100311 | JQ739411 |
| *Pinus heldreichii* | Phe02.100311 | KC136141 |
| *Pinus heldreichii* | Phe03.100311 | KC136142 |
| *Pinus heldreichii* | Phe04.100311 | KC136143 |
| *Pinus heldreichii* | Phe05.100311 | KC136144 |
| *Pinus heldreichii* | Phe06.011012 | KC136145 |
| *Pinus heldreichii* | Phe07.011012 | KC136146 |
| *Pinus heldreichii* | Phe08.011012 | KC136147 |
| *Pinus pinea* | Ppi01.100311 | JQ739412 |
| *Pinus pinea* | Ppi02.100311 | KC136148 |
| *Pinus pinea* | Ppi03.100311 | KC136149 |
| *Pinus pinea* | Ppi04.100311 | KC136150 |
| *Pinus pinea* | Ppi05.100311 | KC136151 |
| *Pinus pinea* | Ppi06.011012 | KC136152 |
| *Pinus pinea* | Ppi07.011012 | KC136153 |
| *Pinus pinea* | Ppi08.011012 | KC136154 |
| *Pinus nigra* | Pn01.100311 | JQ739413 |
| *Pinus nigra* | Pn02.100311 | KC136155 |
| *Pinus nigra* | Pn03.100311 | KC136156 |
| *Pinus nigra* | Pn04.100311 | KC136157 |
| *Pinus nigra* | Pn05.100311 | KC136158 |
| *Pinus nigra* | Pn06.011012 | KC136159 |
| *Pinus nigra* | Pn07.011012 | KC136160 |
| *Pinus nigra* | Pn08.011012 | KC136161 |
| *Pinus eldarica* | Pe01.120311 | JQ739414 |
| *Pinus eldarica* | Pe02.120311 | KC136162 |
| *Pinus eldarica* | Pe03.120311 | KC136163 |
| *Pinus eldarica* | Pe04.120311 | KC136164 |
| *Pinus eldarica* | Pe05.120311 | KC136165 |
| *Pinus eldarica* | Pe06.011012 | KC136166 |
| *Pinus eldarica* | Pe07.011012 | KC136167 |
| *Pinus eldarica* | Pe08.011012 | KC136168 |
| *Pinus brutia* | Pb01.100311 | JQ739415 |
| *Pinus brutia* | Pb02.100311 | KC136169 |
| *Pinus brutia* | Pb03.100311 | KC136170 |
| *Pinus brutia* | Pb04.100311 | KC136171 |
| *Pinus brutia* | Pb05.100311 | KC136172 |
| *Pinus brutia* | Pb06.011012 | KC136173 |
| *Pinus brutia* | Pb07.011012 | KC136174 |
| *Pinus brutia* | Pb08.011012 | KC136175 |
| *Pinus halepensis* | Pha01.100311 | JQ739416 |
| *Pinus halepensis* | Pha02.100311 | KC136176 |
| *Pinus halepensis* | Pha03.100311 | KC136177 |
| *Pinus halepensis* | Pha04.100311 | KC136178 |
| *Pinus halepensis* | Pha05.100311 | KC136179 |
| *Pinus halepensis* | Pha06.011012 | KC136180 |
| *Pinus halepensis* | Pha07.011012 | KC136181 |
| *Pinus halepensis* | Pha08.011012 | KC136182 |
| *Pinus peuce* | Ppe01.100311 | JQ739417 |
| *Pinus peuce* | Ppe02.100311 | KC136183 |
| *Pinus peuce* | Ppe03.100311 | KC136184 |
| *Pinus peuce* | Ppe04.100311 | KC136185 |
| *Pinus peuce* | Ppe05.100311 | KC136186 |
| *Pinus peuce* | Ppe06.011012 | KC136187 |
| *Pinus peuce* | Ppe07.011012 | KC136188 |
| *Pinus peuce* | Ppe08.011012 | KC136189 |
| *Pinus sylvestris* | Psy01.100311 | JQ739418 |
| *Pinus sylvestris* | Psy02.100311 | KC136190 |
| *Pinus sylvestris* | Psy03.100311 | KC136191 |
| *Pinus sylvestris* | Psy04.100311 | KC136192 |
| *Pinus sylvestris* | Psy05.100311 | KC136193 |
| *Pinus sylvestris* | Psy06.011012 | KC136194 |
| *Pinus sylvestris* | Psy07.011012 | KC136195 |
| *Pinus sylvestris* | Psy08.011012 | KC136196 |
| *Pinus brutiaxhalepensis* | Pbh01.120311 | JQ739416 |
| *Pinus brutiaxhalepensis* | Pbh02.120311 | KC136197 |
| *Pinus brutiaxhalepensis* | Pbh03.120311 | KC136198 |
| *Pinus brutiaxhalepensis* | Pbh04.120311 | KC136199 |
| *Pinus brutiaxhalepensis* | Pbh05.120311 | KC136200 |
| *Pinus brutiaxhalepensis* | Pbh06.120311 | KC136201 |
| *Pinus brutiaxhalepensis* | Pbh07.120311 | KC136202 |
| *Pinus brutiaxhalepensis* | Pbh08.120311 | KC136203 |
| *Pinus brutiaxhalepensis* | Pbh09.120311 | KC136204 |
| *Pinus brutiaxhalepensis* | Pbh10.120311 | KC136205 |
